# Diel flight activity of wild-caught *Anopheles farauti* (*s.s.*) and *An. hinesorum* malaria mosquitoes from northern Queensland, Australia

**DOI:** 10.1186/s13071-018-3271-0

**Published:** 2019-01-22

**Authors:** Giles E. Duffield, Dominic J. Acri, Gary F. George, Aaron D. Sheppard, Nigel W. Beebe, Scott A. Ritchie, Thomas R. Burkot

**Affiliations:** 1 Department of Biological Sciences and Eck Institute for Global Health, Galvin Life Science Center, University of Notre Dame, Notre Dame, IN 46556 USA; 2 University of Queensland, School of Biology, St Lucia, Queensland Australia; 3 CSIRO, Dutton Park, Queensland Australia; 4 Australian Institute of Tropical Health and Medicine, James Cook University, Cairns, Queensland Australia

**Keywords:** Mosquitoes, *Anopheles farauti*, *Anopheles hinesorum*, Circadian clock, Diel rhythm, Locomotor activity, Malaria

## Abstract

**Background:**

Species in the *Anopheles farauti* complex are major malaria vectors in the Asia Pacific region. Anopheline mosquitoes exhibit circadian and diel rhythms in sugar- and blood-feeding (biting), flight activity, oviposition, and in some species, a short-lived dusk/early night associated swarming behaviour during which mating occurs. A behavioural study of wild-caught mosquitoes from Queensland, Australia was conducted to investigate the differences in diel rhythmic flight activity between two cryptic species in several reproductive states.

**Results:**

The 24-hour flight activity of individual adult female mosquitoes under light:dark cycle conditions were monitored with a minute-to-minute time resolution using an infrared beam break method. Mosquitoes were analyzed for reproductive state (insemination and parity) and identified to species [*An. farauti* (*s.s.*) Laveran and *An. hinesorum* Schmidt] by PCR analysis. We compared daily total flight activity, timing of activity onset, the peak in early nocturnal activity, and patterns of activity during the scotophase (night). Species-specific differences between *An. farauti* and *An. hinesorum* were observed. Compared to *An. farauti*, *An. hinesorum* had an earlier onset of dusk activity, an earlier peak in nocturnal activity, and a higher level of activity at the onset of darkness. Small differences between species were also observed in the pattern of the dusk/early-night bouts of activity. A second nocturnal peak in inseminated nulliparous *An. hinesorum* was also observed during the middle of the scotophase.

**Conclusions:**

The behavioural differences between these two sympatric species of the *An. farauti* complex might contribute to subtle differences in habitat adaptation, the timing of host-seeking and/or sugar-feeding activity. This study provides baseline data for analysis of populations of mosquitoes from other geographical regions where these species are malaria vectors, such as in the Solomon Islands and Papua New Guinea. This is important as selective pressures due to long-term use of indoor residual spraying of insecticides and insecticide-treated bed nets are shifting the nocturnal profile of biting behaviour of these vectors to earlier in the night.

**Electronic supplementary material:**

The online version of this article (10.1186/s13071-018-3271-0) contains supplementary material, which is available to authorized users.

## Background

Mosquitoes of the *Anopheles farauti* complex (*An. punctulatus* group) are major vectors of human malaria in Indonesia, Papua New Guinea (PNG), the Solomon Islands, Vanuatu and, previously, Australia. Populations of these mosquitoes remain in northern Australia, where malaria was once endemic [1]. The first recorded outbreak of malaria in Australia was in 1843 at Port Essington, Northern Territory [2], although it is likely that the indigenous human population of Australia were exposed to malaria prior to European settlement. Outbreaks continued until the mid 1960s, including the largest Australian civilian outbreak of *P. vivax* in 1942 at Cairns, Queensland, affecting over 700 people [2, 3]. *Anopheles farauti* (*s.l*.), or the *An. farauti* complex, was the purported primary vector during this epidemic [4, 5]. Measures implemented to control malaria included modification of mosquito habitats, swamp drainage to kill larvae, pyrethrum spraying to control adult mosquitoes, and the distribution of mosquito nets [6]. Outbreaks of malaria were common until the latter half of the 20th Century; the most recent notable case occurred in 2002 at Noah Beach, close to Cairns, with transmission of *P. vivax*, and where *An. farauti* (*s.s.*) was identified as the vector [7]. Therefore, the potential exists for malaria transmission to reoccur in Australia should malaria parasites be introduced by the arrival of infected persons or the transportation of infected mosquitoes, most likely from nearby PNG and the Solomon Islands.

The *An. farauti* complex is comprised of eight morphologically indistinguishable species, three of which are common in northern Australia: *Anopheles farauti* (*s.s.*) Laveran (herein referred to as *An. farauti*), *An. hinesorum* Schmidt (herein referred to as *An. hinesorum*) and *An. torresiensis* [1]. Although these species are morphologically indistinguishable, they exhibit habitat and behavioural differences. *Anopheles farauti* is a coastal species and larvae are adapted to brackish water [8–11]. *Anopheles hinesorum* is also readily found in coastal areas, but is also found inland, including highlands, and is adapted to cooler climates [1, 10, 12]. Accordingly, the two species are sympatric in some but not all locations. *Anopheles farauti* and *An. hinesorum* are both anthropophagic in PNG; however, *An. hinesorum* demonstrates geographical variations in host preferences, being predominantly zoophagic in the Solomon Islands [4, 11].

The majority of information regarding *An. farauti* and *An. hinesorum* behaviour is derived from studies in PNG and the Solomon Islands, where they are malaria vectors [10, 11, 13–16]. The role of *An hinesorum* in the transmission of malaria is more complex. Whereas *An hinesorum* is a major malaria vector in PNG, limited human feeding occurs in the Solomon Islands with only a single sporozoite-infected specimen collected in the Western Province [17]. Although there is little evidence that *An. hinesorum* has been a vector in Queensland, Australia [10, 11], its current vector status in PNG and the potential for cold-adaptation to develop, means there is a high priority to study species-specific behaviours of this vector in Australia.

Efforts to control the vector capacity of the *An. punctulatus* group in the Solomon Islands led to behavioural resistance in *An. farauti*, effectively shifting host-seeking and blood-feeding from indoor and outdoor all-night biting to earlier in the evening and outdoors [1, 8, 18–23]. Such changes in the timing of blood-feeding due to selection imposed by insecticide-treated bednets and/or residual spraying has been reported in a range of anophelines, and may limit the efficacy of bednets as a malaria control strategy [24–28]. While human landing catches have been used to assess biting times in areas subjected to insecticidal selective pressure (e.g. regions of the Solomon Islands, PNG, Indonesia and Vanuatu) and in areas with an absence of vector control pressure, it is unclear whether the underlying pattern of flight activity may have been modified in these mosquito populations. We hypothesize that the flight activity profiles of these mosquitoes will parallel their landing catch/biting profiles and hence timing of blood-feeding. Such information on mosquito behaviour may assist in understanding the mechanism of changes in vector behaviour and contribute to our understanding of how to best control the vector in the future. This is especially important in locations where behavioural shifts in biting have occurred, posing complications for the control of malaria transmission [29].

The expression of 24-hour rhythms in mosquitoes at the molecular, physiological and behavioural levels is driven by an interaction between an endogenous circadian clock and the environmental light-dark (LD) cycle [30–36]. Anopheline mosquitoes exhibit predominantly nocturnal patterns of behaviour that include flight activity, swarming and mating, host-seeking/blood-feeding, sugar-feeding, oviposition and pupation [30, 31, 37–40]. Furthermore, many aspects of mosquito biochemistry and physiology are rhythmically regulated, including metabolic detoxification, sensitivity to insecticides, and sensitivity to host odors [34, 38, 41].

Circadian rhythms refer to ~24 h biological processes driven by an endogenous clock, i.e. they persist under constant conditions. Conversely, diel rhythms are 24 h rhythms observed under normal environmental LD cycle conditions. Diel rhythms can either be driven directly by the LD cycle or generated through an interaction between the circadian clock and environmental timing cues [36, 42].

Mosquitoes of the *An. farauti* complex are nocturnal, and their diel landing catch/biting patterns are affected by environmental factors such as moonlight, as well as by gonotrophic age/parity [8, 11, 43–45]. However, there is only a single study that reports on the timing of *An. farauti* (*s.s.*) flight activity, specifically examining its response to differing photoperiods [46, 47]. Although little is known about the diel flight activity patterns of *An. farauti* complex mosquitoes, it is plausible that they are influenced by season, reproductive state and age, as observed in a range of mosquito species [8, 37, 40, 48, 49]. Therefore, exploring the effect of gonotrophic age on diel rhythms in populations of two major malarial vectors may provide useful information that would not be obvious from comparing species alone. Here we report the results of our investigation of diel patterns of spontaneous flight activity comparing the reproductive states of two sympatric species of the *An. farauti* complex in Australia for differences in their temporal profiles of flight activity.

## Methods

### Mosquito collections

*Anopheles farauti* (*s.s.*) and *An. hinesorum* mosquitoes were collected overnight at the same site in northern Queensland, Australia by CO_2_ + octenol-baited passive traps [50] near a mangrove forest at McGregor Road and Dunne Road, Smithfield, QLD 4878, Australia (S16°49'30"S, 145°42'21"E) at 9 m above sea level on October 17 and 23, and November 26, 2014. To reduce metabolic stress in caught mosquitoes, honey-impregnated filter paper was provided and high humidity was maintained by a moist sponge set inside the passive trap. Traps were set during the late afternoon and collected early the next morning. Captured adult female mosquitoes were transported to the laboratory and immediately transferred to the locomotor activity-recording device. Species and reproductive state of each mosquito was assessed after recording activity for at least 4 days.

### Locomotor activity recording

**Fig. 1:**
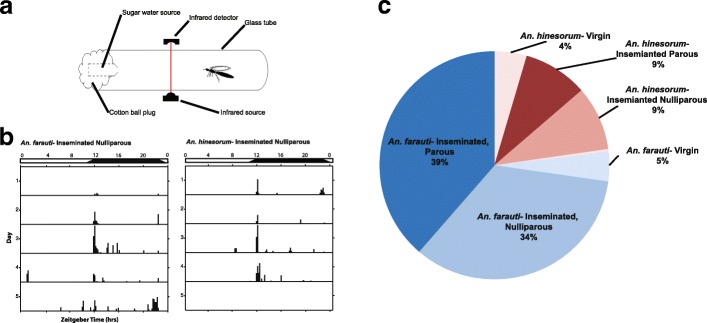
Behavioural analysis of flight activity in two sympatric species of wild-caught *An. farauti* complex mosquitoes. **a** Diagram of LAM25 unit to record individual mosquito activity by continuous infrared beam-break monitoring. **b** Representative actograms from individual *An. farauti* and *An. hinesorum* female mosquitoes. Days are reported on vertical axis and sequential 24 h periods are shown on the horizontal axis. Zeitgeber time (ZT) with ZT12 being the time of lights off, ZT10.5 the start of the 1.5 h dusk transition, and ZT0 the end of the 1.5 h dawn transition and 12 h after the onset of night. Flight activity presented in 6 min bins. Day and night are indicated by horizontal white/black bars. **c** Distribution of proportions of experimental mosquitoes by species and reproductive status

Mosquito flight activity was measured with a Locomotor Activity Monitor 25 (LAM 25) system (TriKinetics, Waltham, MA, USA) by placing individual mosquitoes in 25 × 150 mm clear glass tubes with access to 25% honey or 20% sucrose provided *ad libitum*. Flight activity per minute was monitored by recording breaks in a series of infrared beams (Fig. 1a). LAM 25 units were placed in a light-controlled box on a ~12/12 h LD cycle adjusted to local time civil sunset and sunrise (~10.5 h full light, ~10.5 h darkness, and 90 min dawn and 90 min dusk transitions) with full light in the LAM 25 at 248 lux. Figure 1b shows output of an ‘actogram’ (activity pattern) for an individual mosquito of each species, plotted on a new line for each 24 h day. The height of each bar indicates the intensity of mosquito flight activity in each 6 min time bin, i.e. how many beam break events were detected per unit time. Total duration of the ‘light’ phase of the LD cycle (i.e. duration of full light plus dim light) was set to be equivalent to the ambient conditions when mosquitoes were caught in the field, including twilight, and corroborated from national meteorological information; 13 h 45 min (18th and 24th October collections) and 13 h 19 min (27th November collection). Full light and dawn/dusk transitions were provided by alternate white LED strips (60 per meter; warm white, type 3528; and cool white, type 5050) mounted on the lid of the box. Time of day was reported in 24 h Zeitgeber time (ZT) with ZT12.0 defined as time of lights-off under the LD cycle. The start of the dusk transition was defined as ZT10.5 or ZT10:30. Light intensities at the lower and higher levels of the LAM unit were measured in lux during the dusk cycle: At ZT10:40 light was 69–114 lx; at ZT10:50, 60–100 lx; at ZT11:00, 46–78 lx; at ZT11:10, 33–54 lx; at ZT11:20, 19–31 lx; at ZT11:30, 8.2–14.3 lx; at ZT11:40, 3.1–5.2 lx; at Z11:50, 0.8–1.4 lx; and at ZT11:55, 0.2– 0.33 lx. The reverse occurred for the dawn cycle, which started at ZT22:45 and ended at ZT0:15 (full lights on) (October collections) and started at ZT22:19 and ended at ZT23:50 (full lights on) (November collection), and reflects the 26 min shorter ambient photoperiod between collections. The light/humidity-proof box was kept inside a temperature-controlled insectary. The box was provided with cups containing water and a sponge to facilitate a high level of humidity throughout the experiment. Mosquitoes were maintained at 26 ± 0.5 °C and 80 ± 10% relative humidity, measured using a HOBO datalogger (Onset, Bourne, MA, USA). Mosquito flight activity was monitored for 4–5 days, depending upon survivorship of mosquitoes. Locomotor flight activity was viewed in actogram format using ClockLab v.2.61 analysis program (Actimetrics, Wilmette, IL, USA). A total of 101 female mosquitoes were assayed in three separate trials.

### Analysis of reproductive state and species

At the end of the flight activity assays, mosquitoes were removed from the LAM unit. Reproductive organs were dissected from CO_2_ anesthetized mosquitoes, under a light microscope; spermatheca were examined for spermatozoa, and parity status determined by examining ovaries for the presence (or absence) of tracheole skeins on air-dried slides [51, 52]. Immediately following dissection of a mosquito, the remaining carcass was fixed in 100% ethanol. Fixed mosquitoes were processed at the University of Queensland for genotyping. Genomic DNA was extracted using a salt extraction method [53] and then identified to species by a species-specific PCR-RFLP of the ITS2 locus [54].

### Diel (LD cycle) activity analysis

Flight activity profiles were created for each species and by reproductive status and parity. Mosquitoes were classified as either inseminated nulliparous, inseminated parous, or virgin. Activity was visualized in one minute and one-hour bins to analyze for time-specific differences between species and between species in each of the reproductive/age categories. For each group, the percent accumulation of daily activity over the 24 hour period was also calculated, starting at the onset of the daytime (ZT0) as 0%.

The average onset of flight activity was determined from the activity data recorded by the LAM 25 unit for each mosquito with at least 3 days of flight activity. In some cases either the first or last days were excluded where behaviour was absent or erratic (this occurred in 26 mosquitoes, resulting in a total of 38 days that were excluded). Activity onset was defined as the first minute followed by two consecutive minutes between ZT11 and ZT13 with flight activity of at least one infrared beam break per minute. In some instances, no such continuous block of activity was recorded and those days (*n* = 17 days across 15 mosquitoes) were therefore excluded from the determination of the average daily onset for that mosquito. Accordingly, data for each mosquito was derived from a minimum of 3 days and a maximum of 5 days. Maximum peak of flight activity was determined from activity data recorded by the LAM 25 unit for each mosquito. The data were first smoothed into 5 min running averages, and the average peak value across all days (≥ 3 days) for each animal was determined. The maximum peak was defined as the minute between ZT11 and ZT13 with the highest infrared beam breaks per minute. In days where multiple minutes during this period had equally high activity, the first maximum peak that occurred was considered the peak.

The activity data were also smoothed using a 30 min running average, transformed by Z-score normalization, and analyzed at a 1 min resolution for differences in activity between species across the 24 h period. A more focused analysis between ZT11 and ZT13 was also performed in which the data were smoothed using a 5 min running average, Z-scored, and also analyzed at the level of 1 min sampling (i.e. minute-to minute).

### Statistical analysis

Statistical analysis and graphing were performed using Sigma-Plot 12 (Systat Software, Chicago, IL, USA) and GraphPad Prism 5 (Prism, La Jolla, CA, USA). Non-parametric statistical analyses were used when the Shapiro-Wilk normality test failed (*P* < 0.05) or data were cube-root or square-root transformed to correct for non-normal distributions to allow for parametric analysis. For data that passed a Shapiro-Wilk normality test, a one-factor ANOVA or Student’s t-test was performed to evaluate differences between groups. For consistency, in comparing the onset times between reproductive states and species-specific reproductive groups data that did not pass normality test, even when square- and cube-roots of values were analyzed, non-parametric tests were utilized. Due to the low capture rate of virgin mosquitoes, virgin female groups were excluded from statistical analyses when based on reproductive state. Statistical significance was at the level of *P* < 0.05.

## Results

### Profile of the wild-caught mosquitoes used in the activity analysis

The dominant reproductive status of wild-caught female *An. farauti* and *An. hinesorum* was inseminated-nulliparous (Fig. 1a, c). Overall, activity data was obtained for 101 mosquitoes, of which 44 were successfully assessed for both spermatheca status and ovary status. Ninety-one percent of the sampled females were inseminated (spermatozoa positive; *n* = 40) and 9% were virgin (spermatozoa negative; *n* = 4), and 48% (*n* = 21) had previously oviposited (were parous), leaving 43% (*n* = 19) that were nulliparous. No mosquitoes were identified as gravid. Molecular analysis revealed that 69 (68%) of anophelines were *An. farauti* (*s.s*.) and 32 (32%) were *An. hinesorum.* Activity for 3–5 days (recorded as beam break counts) (Fig. 1a) revealed distinct diel nocturnal flight activity (Fig. 1b).

Analysis of flight activity for all mosquitoes collected: *Anopheles farauti* and *An. hinesorum* mosquitoes both showed daily nocturnal rhythms of flight activity with a pronounced activity peak during the early night. There were no statistically significant species differences in total counts of flight activity, possibly due to small sample sizes and large variance. However, these trends suggest a higher level of activity in *An. hinesorum* across all reproductive states compared to *An. farauti*. *Anopheles farauti* females (*n* = 69) had a median daily activity of 133 beam break counts per day (counts), while *An. hinesorum* (*n* = 32) had a median count of 218 (Mann-Whitney test, *U*_(99)_ = 910.50, *P* = 0.158), 85 counts more than *An. farauti*.

Analysis of total daily activity for mosquitoes of known gonotrophic state: Inseminated nulliparous *An. farauti* (*n* = 15) had a median daily activity of 198 counts, while inseminated nulliparous *An. hinesorum* (*n* = 4) had a median count of 408, 210 counts more than *An. farauti* (*U*_(17)_ = 17.00, *P* > 0.05). Inseminated parous *An. farauti* (*n* = 17) had a median daily activity of 192 counts, while inseminated parous *An. hinesorum* (*n* = 4) had a median count of 475, 283 more than *An. farauti* (*U*_(19)_ = 15.00, *P* = 0.097). Virgin *An. farauti* (*n* = 2) had a median daily activity of 188 counts, while virgin *An. hinesorum* (*n* = 2) had a median count of 219, 31 counts more than *An. farauti*.

**Fig. 2:**
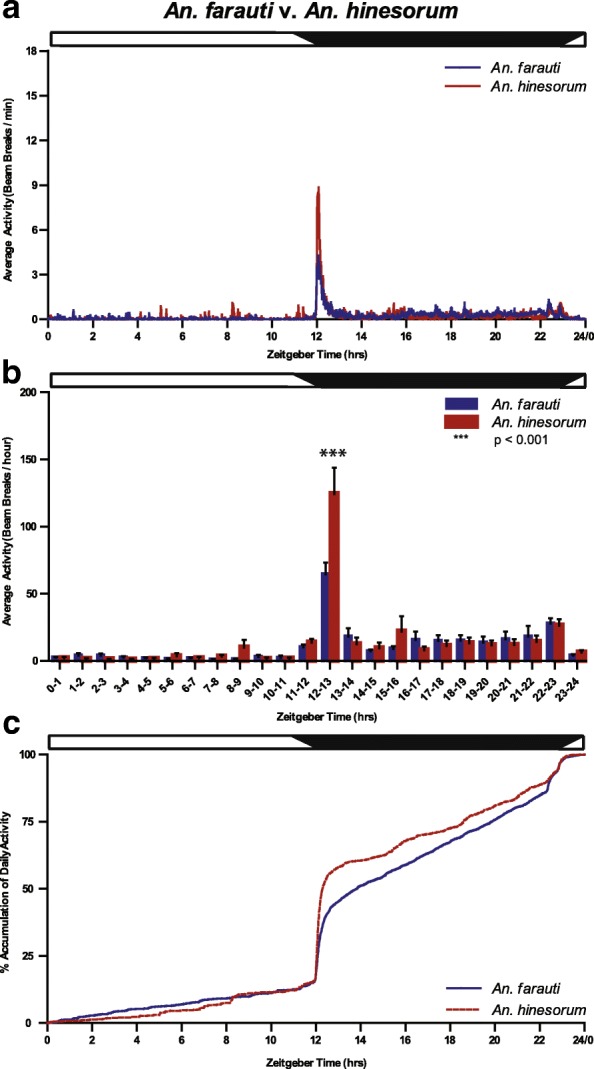
Average and accumulated activity over the 24 h day in *An. farauti* and *An. hinesorum* mosquitoes. Twenty-four hour distribution of mosquito flight activity measured by infrared beam breaks of *An. farauti* (blue) and *An. hinesorum* (red) females regardless of reproductive status under LD cycle conditions. **a** Activity counts per minute (mean ± SEM). **b** Total activity within each hourly time bin (mean ± SEM). **c** Accumulation of daily total mosquito activity. Statistical analysis of accumulation data performed at 50 and 75% of total daily activity (Additional file 3: Figure S3). Data lines represent average percent measures. Zeitgeber times as for Fig. 1 legend. Day and night are indicated by horizontal white/black bars. Two-factor repeated measures ANOVA was performed followed by Tukey’s *post-hoc* tests, ***P < 0.001

**Fig. 3:**
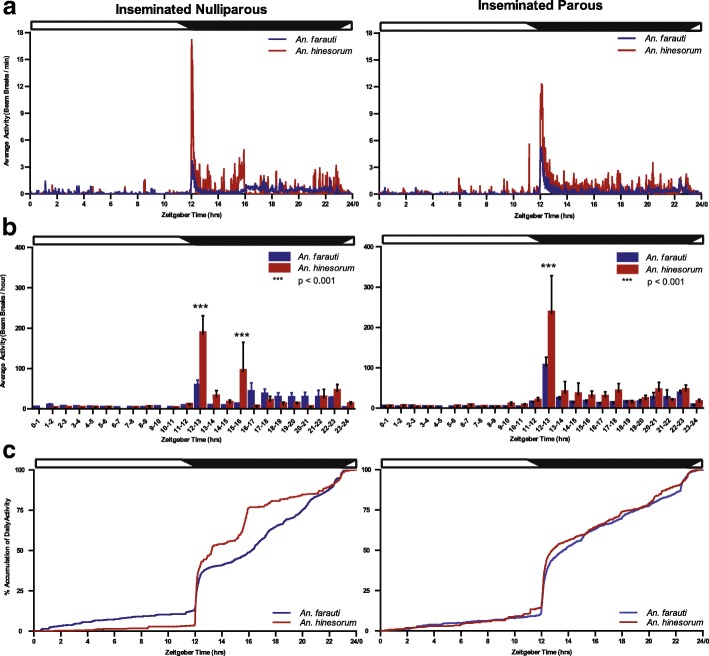
Flight activity profiles for *An. farauti* and *An. hinesorum* mosquitoes according to reproductive status. Twenty-four hour distribution of mosquito flight activity measured by infrared beam breaks of *An. farauti* (blue) and *An. hinesorum* (red) females of each reproductive state under LD cycle conditions at **a** minute and **b** hour resolutions. Hour resolutions are mean ± SEM of total activity within each hourly time bin. **c** The accumulation dynamics of daily total mosquito activity. Zeitgeber times as for Fig. 1 legend. Day and night are indicated by horizontal white/black bars. Two-factor repeated measures ANOVA was performed followed by Tukey’s *post-hoc* tests, ***P < 0.001

Analysis of differences in the pattern of flight activity across the diel between species: Flight activity data were arranged into 1-hour bins to analyse the hourly pattern of flight across the diel (Figs. 2 and 3). A pronounced species difference in the pattern of activity was noted following the onset of night at ZT12–13 (Fig. 2); although there was no significant main effect of species, the effect of ZT diel time and the interaction between species and time were significant (two-way repeated measures ANOVA: effect of species, *F*_(1,2039)_ = 0.87, *P* = 0.354; effect of ZT, *F*_(23,2039)_ = 34.16, *P* < 0.001; interaction, *F*_(23,2039)_ = 4.09, *P* < 0.001; Fig. 2b). The activity levels in *An. hinesorum* at ZT12–13 were elevated relative to *An. farauti*.

Analysis of flight activity with regard to gonotrophic state: a pronounced species difference in flight activity for both nulliparous and parous groups was observed during early night (ZT12–13), and additionally in the inseminated nulliparous group, a species difference was detected 4 h into the scotophase (ZT15–16) (nulliparous: species, *F*_(1,455)_ = 0.83, *P* = 0.375; ZT, *F*_(23,455)_ = 6.99, *P* < 0.001; interaction, *F*_(23,455)_ = 2.99, *P* < 0.001; parous: species, *F*_(1,502)_ = 3.08, *P* = 0.095; ZT, *F*_(23,502)_ = 14.60, *P* < 0.001; interaction, *F*_(23,502)_ = 2.28, *P* < 0.001; Fig. 3b). At these two specific times of the night, the activity levels in *An. hinesorum* were elevated relative to *An. farauti*.

When the data were examined to specifically compare reproductive states within each species, higher levels of activity were detected in parous *An. farauti* at ZT12–13. Conversely higher levels were observed in nulliparous *An. farauti* during the middle of the scotophase at ZT16–17 (reproductive state, *F*_(1,767)_ = 0.00, *P* = 0.958; ZT, *F*_(23,767)_ = 9.40, *P* < 0.001; interaction, *F*_(23,767)_ = 1.58, *P* = 0.042; Additional file 1: Figure S1b). However, no significant differences were observed between reproductive groups for *An. hinesorum* (reproductive state, *F*_(1,191)_ = 0.43, *P* = 0.535; ZT, *F*_(23,191)_ = 9.15, *P* < 0.001; interaction, *F*_(23,191)_ = 0.50, *P* = 0.973; Additional file 1: Figure S1b).

Comparison of the two species using the smaller cohort of mosquitoes for which insemination and parity could be determined revealed a pronounced species difference at ZT15–16 as well as at ZT12–13 (species, *F*_(1,1055)_ = 2.54, *P* = 0.119; ZT, *F*_(23,1055)_ = 22.33, *P* < 0.001; interaction, *F*_(23,1055)_ = 4.30, *P* < 0.001; Additional file 2: Figure S2). While the higher activity seen for *An. hinesourum* at ZT12–13 is consistent with the larger cohort of mosquitoes (Fig. 2), the higher activity at ZT15–16 is a feature consistent with the nulliparous subgroup (Fig. 3).

The pattern of accumulated flight activity over 24 h (measured over 3–5 days) was compared between the two species to determine when mosquitoes had accumulated a given amount of their daily total flight activity (Figs. 2c and 3c). A significant species difference was found in percent accumulation at the time at which *An. farauti* and *An. hinesorum* reached 75% accumulation of activity (*U*_(83)_ = 555.00, *P* = 0.031), with *An. hinesorum* reaching this point earlier. *Anopheles hinesorum* shows similar accumulation over the light phase of the LD cycle (day) (ZT0 to ZT12), but differentiates during the dark phase of the cycle (scotophase) (Fig. 2c). While not significant, the *An. hinesorum* mean value also reached 50% accumulation of activity before *An. farauti* (*t*_(83)_ = 0.747, *P* = 0.457) (Additional file 3: Figure S3). Furthermore, no significant reproductive state differences were detected at these two accumulation thresholds (Fig. 3c).

### Times of onset and maximum peak of dusk/early night flight activity

**Fig. 4:**
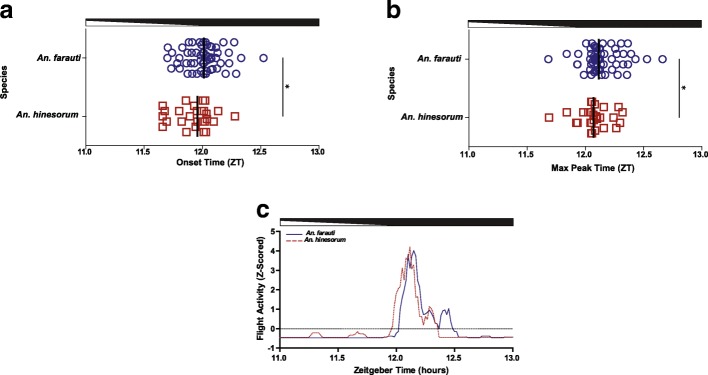
Onset and peak of dusk/early night-related flight activity in *An. farauti* and *An. hinesorum* mosquitoes. Differences between *An. farauti* (blue) and *An. hinesorum* (red) in time of dusk related onset of flight activity and peak of flight activity: **a** time of onset of activity, **b** time of peak of activity, and **c** a representative Z-score plot of activity. Median values (lines) and individual mosquitoes (*An. farauti*, circles; *An. hinesorum*, squares) in Zeitgeber time (ZT, h). Dusk transitions are indicated by horizontal white/black bars. Mann-Whitney U-tests were performed, *P < 0.05. **c** Representative activity plots for individual mosquitoes from each species, demonstrating a delayed onset and delayed peak of activity for *An. farauti* compared to *An. hinesorum*

Species- or reproductive state-specific differences in the time of activity onset and the maximum peak of elevated dusk-related flight activity, key features of the diel activity profile, were investigated. A significant difference was revealed in activity onset times between *An. farauti* and *An. hinesorum* (*U*_(83)_ = 547.00, *P* = 0.033) (Fig. 4a). *Anopheles farauti* commenced activity at a median time of ZT12:01, while *An. hinesorum* activity onset was 3 min earlier at a median time of ZT11:58. The mean onset of the *An. farauti* mosquitoes was ZT12:01, while the *An. hinesorum* mean activity onset commenced 4.8 min earlier at ZT11:56 (Table 1). No significant differences were observed in the time of activity onset among reproductive state groups (inseminated parous *versus* inseminated nulliparous, *U*_(38)_ = 158.0, *P* = 0.268) or species-specific reproductive state groups (Kruskal-Wallis one-way ANOVA, *H*_(3)_ = 3.98, *P* = 0.264) (Additional file 4: Figure S4a, Tables 2 and 3).

**Table 1:** Mean ± SEM in Zeitgeber time (ZT) for onset of activity and maximum peak values in *An. farauti* and *An. hinesorum* mosquitoes

Species	*Anopheles farauti*	*Anopheles hinesorum*
	(*n* = 58)	(*n* = 27)
Onset (ZT)	12:01 ± 0:01	11:56 ± 0:02
Maximum peak (ZT)	12:09 ± 0:01	12:05 ± 0:02

**Table 2:** Mean ± SEM in Zeitgeber time (ZT) for onset of activity and maximum peak values for both *An. farauti* and *An. hinesorum* combined and comparing three reproductive states

Reproductive state	Inseminated, parous	Inseminated, nulliparous	Virgin
	(*n* = 21)	(*n* = 19)	(*n* = 4)
Onset (ZT)	11:58 ± 0.02	12:02 ± 0:02	12:02 ± 0:03
Maximum peak (ZT)	12:07 ± 0:01	12:09 ± 0:02	12:12 ± 0.04

**Table 3:** Mean ± SEM in Zeitgeber time (ZT) for onset of activity and maximum peak values for *An. farauti* and *An. hinesorum* and across three reproductive states

Species	*Anopheles farauti*			*Anopheles hinesorum*		
Reproductive state	Inseminated, parous	Inseminated, nulliparous	Virgin	Inseminated, parous	Inseminated, nulliparous	Virgin
	(*n* = 17)	(*n* = 15)	(*n* = 2)	(*n* = 4)	(*n* = 4)	(*n* = 2)
Onset (ZT)	11:58 ± 0:02	12:04 ± 0:03	12:07 ± 0:04	11:57 ± 0:05	11:53 ± 0:05	11:59 ± 0.03
Maximum peak (ZT)	12:06 ± 0:01	12:09 ± 0:04	12:12 ± 0:07	12:11 ± 0:04	12:09 ± 0:04	12:12 ± 0:07

A significant difference between *An. farauti* and *An. hinesorum* maximum peak in flight activity per minute (with a 5 min running average) between ZT11 and ZT13 was found (*U*_(83)_ = 565.5, *P* = 0.040) (Fig. 4b). The median peak time in *An. farauti* occurred at ZT12:07, while the median peak time in *An. hinesorum* was 3 min earlier at ZT12:04. Similarly, the mean peaks for *An. farauti* and *An. hinesorum* were at ZT12:09 and ZT12:05, respectively, revealing a 4.2 min difference (Table 1). Significant differences were not found for the time of the maximum peak between reproductive states (inseminated parous *versus* inseminated nulliparous, *U*_(38)_ = 187.5, *P* = 0.753) or species-specific reproductive state groups (*H*_(3)_ = 1.24, *P* = 0.743) (Additional file 4: Figure S4b, Tables 2 and 3). The earlier onset and earlier maximum peak of activity in *An. hinesorum* can be seen in a Z-scored plot of two individual representative mosquitoes (Fig. 4c). See Tables 1, 2 and 3 for species and reproductive group-specific mean ± SEM activity onset and maximum peak values.

**Fig. 5:**
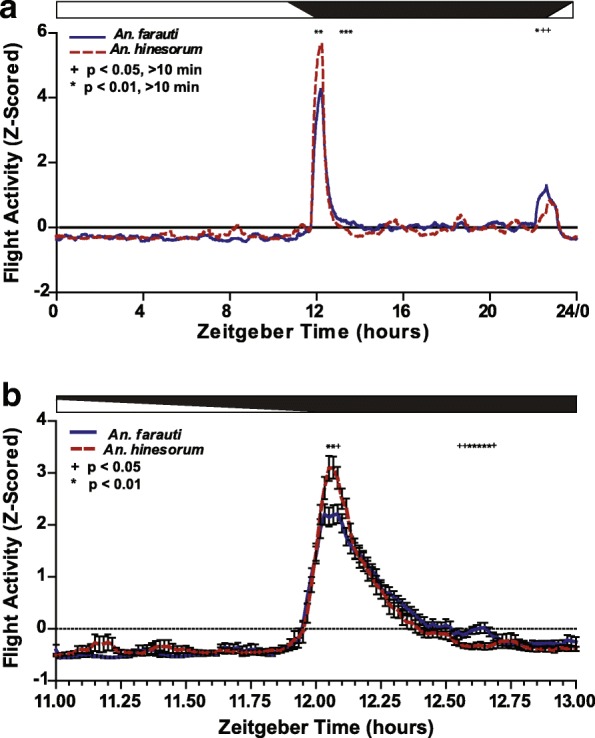
Z-scored flight activity shows multiple time differences between *An. farauti* and *An. hinesorum* mosquitoes at the onset of night and during other parts of the scotophase. **a** Flight activity of *An. farauti* (blue) and *An. hinesorum* (red) over 24 h under normal LD conditions. Values are mean of Z-scored activity. Day and night are indicated by horizontal white/black bars. **b** Dusk and early night-related flight activity. Values are mean ± SEM of Z-scored activity between ZT11 and ZT13 under normal LD cycle conditions. Time shown in Zeitgeber time (ZT) decimal format. Dusk progression shown by horizontal white/black bar. Student’s t-tests were performed, +*P* < 0.05, *P < 0.01

To determine if the dusk/early night related flight activity differed between *An. farauti* and *An. hinesorum* in their temporal dynamics, we analyzed the build-up and decline of daily activity with the activity of each individual animal first smoothed to 30 min and Z-scored; species differences were reported when ≥ 10 consecutive minutes were significantly different. As shown in Fig. 5a, there is a relative increase in mosquito activity around the time of lights-off (i.e. ZT12:00) between ZT11:55 to ZT12:15 in *An. hinesorum* (*n* = 27) relative to *An. farauti* (*n* = 58) (t-tests, *P* < 0.01). Additionally, there was a significant decrease in mosquito activity after the peak of activity between ZT13:39 and ZT14:09 (t-tests, *P* < 0.01). Furthermore, *An. farauti* showed a relative increase in activity later in the night between ZT22:09 and ZT22:39 (t-tests, ZT22:09-ZT22:18, *P* < 0.01; ZT22:19-ZT22:39, *P* < 0.05).

In addition to the 24 h analysis of behaviour, a targeted analysis of dusk/early night associated activity between ZT11 and ZT13 was examined as this is the expected time of onset of behaviours related to migration, sugar-feeding, host-seeking, and in the case of virgins and gravids, respective mating and oviposition activities. The flight activity of each individual animal was smoothed to 5 min and Z-scored. Figure 5b shows consecutive minutes of significance. *Anopheles hinesorum* showed a relative increase of activity compared to *An. farauti* from ZT12:03-ZT12:05 (t-tests, ZT12:03-ZT12:04, *P* < 0.01; ZT12:05, *P* < 0.05). After the peak of activity, *An. hinesorum* showed a decrease in activity relative to *An. farauti* from ZT12:33 to ZT12:39 (t-tests, ZT12:35-ZT12:38, *P* < 0.01; ZT12:33-ZT12:34, ZT12:39, *P* < 0.05). Analysis of mosquitoes arranged by reproductive group and by both species and reproductive group yielded no significant differences.

## Discussion

The flight activity patterns of two sympatric and cryptic species of the *An. farauti* complex, *An. farauti* (*s.s.*) and *An. hinesorum*, in northern Queensland, Australia were characterized. We investigated the potential temporal differences in behaviour between the species, including total daily activity, accumulation dynamics, and dusk/early night-related flight activity, and their relationship to reproductive state. Both *An. farauti* and *An. hinesorum* exhibited daily nocturnal rhythms of flight activity with a pronounced peak at the onset of night at the end of dusk, and a smaller but obvious peak towards the end of night at dawn. During the remainder of the scotophase, intermittent and often lower level activity was observed. This general profile of 24 h activity is consistent with observations described previously in adult anopheline mosquitoes, including *An. farauti* [35, 37, 47, 55]. Such ‘bimodal’ activity patterns are preserved under constant dark conditions, indicating the underlying circadian clock contributes to the regulation of the behaviour. Furthermore, the bout of intense activity observed occurring at dusk/early night (ZT12–13) is relatively short-lived, being less than 45 min, and matches the length of a similar dusk bout of activity documented in the anopheline mosquitoes in the *An. gambiae* complex and in *An. stephensi* [8, 35, 37, 40, 49, 56].

Diel analysis of flight activity behaviour in the *An. farauti* complex has thus far been limited to a single study of adult female *An. farauti* Laveran [presumed *An. farauti* (*s.s.*)], examined under differing photoperiods [47]. The study by Taylor [47] differs considerably from the present analysis, as it used acoustic recordings to assay flight activity, exposed mosquitoes to abrupt transitions from light to dark and dark to light, and the temporal resolution of analysis was relatively large (1 hour bins only) [47]. Furthermore, it is unclear whether the subjects studied were captured as adults or reared from larvae. In the present study, we attempted to tease apart potential species- and reproductive stage/age-specific differences for two species in the *An. farauti* complex. Wild-caught female mosquitoes were subjected to a LD cycle with dawn and dusk transitions, recorded beam-break activity by minute, and analysed data by hourly and minute-to-minute resolution, thereby allowing for a precise temporal evaluation of behaviour [35].

When the accumulation of flight activity is measured from the start of daytime (ZT0), activity accumulates more rapidly in *An. hinesorum* than *An. farauti*. This separation becomes obvious shortly after the onset of night at ZT12, and when analyzed specifically at 75% accumulation, we see a clear significant difference. This difference is mostly due to the higher level of relative activity expressed by *An. hinesorum* compared to *An. farauti* in the dusk/early night-related bout of intense activity (~ZT12–13). The raw counts within this 1-h time bin show *An. hinesorum* exhibiting levels of activity twice as large as those by *An. farauti*. Interestingly, when we compare dusk/early night flight activity, the higher level of activity occurred for a duration of ~20 min between ZT11:55 and ZT12:15. The lower level of activity, as measured in raw counts or following Z-scored normalization, that occurs later and intermittently throughout the remainder of the scotophase/night is comparable between the two species. This focuses our attention on the dusk/early night related activity and that may relate to one or a combination of natural behaviours observed in *An. farauti* complex mosquitoes, such as host-seeking, and behaviours reported in other anophelines, such as migration, sugar-feeding, egg laying in gravids, and in virgins, swarming and mating. This differential activity pattern may represent an aspect of behaviour(s) that is elevated in *An. hinesorum*, e.g. increased feeding activity and/or longer distance/duration of flight.

*Anopheles hinesorum* began its nocturnal activity 5 min earlier on average than *An. farauti*, and the maximum peak of activity in *An. hinesorum* was observed to occur 4 min earlier than for *An. farauti*. This apparent ‘shift’ in the activity profile is of particular interest because the differences occur close to ZT12, the onset of night. The intense level and subsequent peak of activity occurring during the early night is the most dominant and consistent feature of the diel profile. This distinct profile may represent a combination of behavioural drives, many of which have been studied in anophelines and in particular members of the *An. gambiae* complex. These include migration, sugar-feeding, host-seeking and biting, all of which commence during twilight and the onset of night [21, 56–59].

As the majority of captured mosquitoes in the study were identified as either nulliparous or parous females, and few were scored as virgin and none scored as gravid, this makes swarming/mating and oviposition unlikely behaviours to be represented in the underlying flight activity drives of mosquitoes observed here. However, swarming and mating in other anophelines occurs during a small temporal window during dusk [60–62], and peak ovipositioning occurs during the first hour of the night [37, 39, 63], both of which are coincident with the peak in flight activity of *An. farauti* complex mosquitoes in the present study.

Due to the reproductive states of the majority of mosquitoes in the study, it is more likely that the intense early nocturnal activity in the flight profile in part reflects other factors, such as movement from resting sites, i.e. daily migration. In *An. gambiae*, dusk/early night is the time when females depart from their daytime resting sites [57–59]. Furthermore, the flight range for *An. farauti* has been estimated to be as much as 1.6 km [64].

Sugar-feeding activity in *An. gambiae* occurs almost exclusively at night and during the dusk/dawn transitions [56]. However, the expression of this diel pattern is dependent upon the reproductive state of the female [56]. Under laboratory conditions, in inseminated *An. gambiae* females that were either prevented from blood-feeding or blood-fed but prevented from ovipositioning, a high level of sugar-feeding activity was observed during the scotophase and with a noticeable peak during late dusk and early scotophase. Therefore, in the present study, it is possible that the nocturnal flight activity profiles of *An. farauti* complex females, and especially the dusk/early night peak, reflects this characteristic sugar-feeding behaviour.

Finally, host-seeking behaviour and the propensity to bite are also likely contributing drives underlying the diel flight activity profiles of *An. farauti* and *An. hinesorum*. Studies of *An. farauti* and to a lesser degree *An. hinesorum* host-seeking behaviour have been undertaken in the Solomon Islands and PNG, primarily using human landing catch (HLC) assays. In many locales, the nocturnal biting profiles for *An. farauti* consistently show a majority of feeding early in the night. For example, in the Solomon Islands (Haleta, Central Province), 82% of mosquitoes were captured between 18:00 and 21:00 h, and only 18% between midnight and 06:00 h [22]. A major peak occurred at 19:00-20:00 h, declining thereafter, and then a small increase in capture rate occurred at the end of the night/dawn between 05:00-06:00 h [21]. In Honiara, Solomon Islands, as much as 84% of *An. farauti* mosquitoes captured by HLCs was during the first hour following sunset [9]. However, in comparison in PNG (Mirap, Madang Province) the shape of the profile is different: an early night peak remains, although a secondary peak occurred between 23:00 h and midnight, i.e. during the middle of the night [21].

A single study reports on the outdoor biting cycle of *An. hinesorum* as compared to *An. farauti* in PNG (Madang and East Sepik provinces) [10]. While *An. hinesorum* exhibited an early biting profile, with 66% activity occurring between 18:00 and 22:00 h, *An. farauti* (*s.s.*) exhibited a uniform biting profile throughout the entire night and with no obvious peaks. Clearly this study reveals differences in the biting cycle between the two species, as well as *An. hinesorum* in this locale being an early night biter.

There are three major factors thought to contribute to the shape of the *An. farauti* host-seeking biting profile, namely long-term exposure to insecticide, outdoor or indoor location of the host and the lunar cycle. In areas where *An. farauti* (*s.s.*) populations have been exposed to residual spraying of DDT and/or introduction of insecticidal treated bednets, mosquitoes show significant shifts in their biting profiles, developing an exaggerated peak in biting during the onset of night, as well as increasing the number of outdoor *versus* indoor biting encounters [1, 8, 18–23]. For example, in the Solomon Islands (Baunasughu, Manibwenta and Maniparegho) prior to exposure to insecticide, indoor biting profiles showed a more uniform pattern during the first half of the night or show peak biting at midnight [20]. Outdoor biting prior to insecticide exposure still showed a peak close to the onset of the night and highest levels of biting occurred during the first half of the night. Following exposure, both indoor and outdoor patterns became similar, with as much as 45% of nocturnal biting events occurring at dusk and the onset of night (18:30–19:30 h) [20]. Finally, moonlight can impact the nocturnal biting cycle of *An. farauti* [8]. In PNG (Maraga, Madang Province), the biting cycle of *An. farauti* showed an initial peak at the onset of night at all times of the lunar cycle. However, on moonless nights, this initial peak was most prominent, followed by a secondary peak occurring before midnight. Under a waxing moon, activity was concentrated in the first half of the night; under a waning moon activity was also highest during the first half of the night, but activity during the second half of the night remained relatively high compared to other phases of the moon. During a full moon, peak biting occurred in the middle of the night.

Relating the natural biting cycles of *An. farauti* and *An. hinesorum* is obviously complex due to these differing environmental factors. However, the location in Queensland, Australia where the mosquitoes were captured is not an area with known exposure to insecticides, mosquitoes were captured outdoors, and the conditions in the experimental chamber when assayed for flight activity were 0 lux at night (i.e. the equivalent to a moonless night). Based on the studies from the Solomon Islands and PNG, and under these environmental conditions in Australia, we might predict that the *An. farauti* (*s.s.*) mosquitoes would express a diel biting profile with a major peak occurring at the end of dusk into the early night, followed by continued lower level host-seeking activity during the first half of the night. In fact, a recent study in northern Queensland reveals *An. farauti* (*s.s.*) during the dry season as exhibiting early night peak biting. Biting was greatest at the onset of night, and while rates declined throughout the scotophase, a secondary peak occurred at 21:00-22:00 h followed by a small increase occurring near to dawn (W. K. Chow, R. D. Cooper, L. Rigby, P. Pickering, M. Lokhorst and N. Beebe, personal communication). Clearly, there is a correlative relationship between the diel cycles of flight activity and host-seeking activity for *An. farauti* (*s.s.*) in Queensland. This includes the intermittent flight activity observed during the scotophase, but especially so at the onset of night when peak outdoor biting occurs, and towards the end of night/dawn when there is a small but distinct rise in both activities. Therefore, it is likely that the diel flight activity profile reflects components of the drive to host seek and blood-feed.

In *An. farauti* there were differences in the flight activity profiles between nulliparous and parous mosquitoes: activity at the onset of night (ZT12–13) was higher in parous group, while conversely, activity was greater during the middle of the night (ZT16–17) in the nulliparous population. It is unclear what this finding means in terms of behavioural drives that may be different between these groups, except that the parous population is predicted to be older. There are known differences in the biting profiles of anophelines, where parous females tend to bite later than nulliparous females [65–68]. However, this phenomenon observed in the field may be associated with gravids ovipositing early in the night, thereby delaying host-seeking activity during the same night. However, it is unclear whether the differences observed in the flight activity profiles between the reproductive states/ages represent intrinsic differences in biting activity.

Thirty minutes after lights off at ~ZT12.5, *An. farauti* exhibited a small but significant additional elevation of activity compared to *An. hinesorum* for 8 min. While the meaning of this differential species activity in terms of natural behaviour is unknown, a similar differential pattern of behaviour was documented for *An. gambiae* and *An. coluzzii* males [35]. Here, the two *An. gambiae* complex species show a separation in their pattern of activity in both the rise in activity after activity onset occurs during dusk, and on the decline of activity following its peak shorty after the onset of night. In that study the authors speculate that this differential pattern of activity represents either earlier swarm-assembly behaviour, or some aspect of pre/post-swarming behaviour(s), e.g. increased sugar-feeding activity and/or longer distance/duration of flight. This hypothesis may equally apply to *An. hinesorum* and *An. farauti*.

Another bout of differential activity was observed approximately an hour after lights off at ~ZT13 and for a longer duration of time (for ≤ 30 min). As this occurs in the scotophase well after the dusk/onset of night-related high intensity bout of activity, this ‘event’ may underlie a natural behaviour distinct from those associated with dusk and the onset of night.

When reproductive status/age is considered, the enhanced level of flight activity at dusk/onset of night in *An. hinesorum versus An. farauti* was conserved in both groups, namely inseminated nulliparous and inseminated parous. Furthermore, a distinct species difference was observed in the inseminated nulliparous group during the middle of the scotophase/night (ZT15–16), in which *An. hinesorum* mosquitoes had a pronounced elevated bout of activity. This revealed a conspicuous spike amongst the relatively low level activity recorded during the remainder of the scotophase. In *An. hinesorum*, flight activity during this hour was not only considerably higher compared to *An. farauti* but was relatively high compared to almost all times of the night excluding the dusk peak. Interestingly, this elevation of flight activity among inseminated nulliparous *An. hinesorum* at ZT15–16 is consistent with a previous study of HLAs of *An. farauti* complex mosquitoes in PNG (Madang and East Sepik provinces) [10]. In this study, biting-landing catches in *An. hinesorum* were higher during the one hour in the middle of the night at midnight as compared to the preceding two hours and proceeding four hours. This variable pattern of landing catches was not observed for *An. farauti*, which was more uniformly distributed during the night. It is unclear whether this is simply a coincidence or whether the flight activity pattern observed here is associated with host-seeking behaviour and subsequent human encounters.

These behavioural data provide novel insights into the temporal regulation of flight activity that are important for ongoing efforts to understand *An. farauti* complex mosquitoes in Australia, the Solomon Islands, PNG, Indonesia and Vanuatu. Historically, northern Australia was malaria-endemic and, while now eliminated, there remains a risk of re-introduction and local transmission due to presence of *An. farauti*, and possibly *An. hinesorum*. As recent as 2002 there was an outbreak of *P. vivax* transmission in the Cairns area of Northern Queensland, illustrating the potential for local malaria transmission following introduction of the malaria parasite by infected people. Thus, the *An. farauti* complex poses a plausible risk for malaria transmission as Australia is surrounded by countries where malaria is endemic [1, 7]. These data also contribute to our understanding of *An. farauti* complex mosquitoes in malaria endemic areas, including the Solomon Islands and Papua New Guinea, where as much as 6% of the human population is infected with *P. falciparum*, *P. vivax*, or *P. malariae* [14, 15]. Furthermore, as the Queensland populations of *An. farauti* and *An. hinesorum* are not routinely exposed to insecticides, these behavioural data can serve as a baseline for comparison with mosquitoes from the Solomon Islands, where the timing of nocturnal biting activity has been shaped by continuing insecticide selective pressure (from insecticide treated bednets and/or residual spraying) [11, 18, 20]. Although *An. farauti* and *An. hinesorum* are morphologically indistinguishable species and are known to reside in slightly different habitats, differences in the timing of their flight behaviour, as proposed in this paper, and in host-seeking behaviour [10, 11], make vector control complicated in the areas in which the two species coexist. Understanding the causative factors underlying differences in flight activity may have real implications for controlling these two species, particularly as the number of recommended interventions expands.

The shifts in *An. farauti* complex biting activity from the middle of the night to earlier in the evening following insecticidal pressure (from insecticide treated bednets and/or indoor residual spraying of insecticides) [20–22], is a function of changes in the flight activity patterns of the mosquitoes, especially for host-seeking females. Further analysis of the flight activity of wild populations of *An. farauti* and *An. hinesorum* that have been exposed to long-term insecticide pressure would allow for this hypothesis to be tested. Such populations exist in regions of the Solomon Islands in particular. Therefore, the present analysis may provide a baseline for comparing populations of species in the *An. farauti* complex.

There are certain limitations to the interpretation of these data as the experimental conditions do not represent true unconstrained flight conditions in the wild. The absence of males and insufficient numbers of virgin females in this study limit our understanding of potential species differences in the timing of mating and swarming activity. In other anophelines, it is the males that first assemble the swarm around dusk into which virgin females then enter and mate [37, 69], and it has been proposed that differential timing of swarming might contribute in part to species-specific segregation of mating in sympatric *An. gambiae* complex mosquitoes [35, 70, 71]. Therefore, the timing of male activity may be more relevant in the context of temporal control of intraspecific mating [35]. However, these data do provide some insight into the precise timing of aspects of flight behaviour in wild populations of predominately inseminated host-seeking mosquitoes (*versus* laboratory-raised colonies). It is therefore possible that additional but subtle differences exist between the two species and reproductive/age categories.

## Conclusions

In conclusion, in this study of *An. farauti* and *An. hinesorum* mosquitoes from northern Queensland, Australia, species and gonotrophic-age related differences were revealed in their circadian/diel-driven flight behaviour. The dusk/early night-related flight activity in *An. hinesorum* females start and peak earlier than in *An. farauti*. The shape of the dusk/early night flight activity also differs between species, with *An. hinesorum* exhibiting a higher level of activity at its peak around ZT12-ZT12.25, and at its decline *An. farauti* exhibited a small elevation of activity later in the night at ~ZT12.5. Then one hour into the night at ~ZT13, the two species show a small divergence in activity. We hypothesize that this differential pattern of activity observed during late dusk/early night represents species-specific differences in the timing and pattern of host-seeking behaviour, sugar-feeding activity, and/or distance/duration of migratory flight. Finally, we observed a peak in activity during the middle of the night at ZT15–16 in the *An. hinesorum* inseminated nulliparous population, which may be related to increased host-seeking behaviour. As *An. farauti* and *An. hinesorum* are major malaria vectors from Indonesia through PNG and the Solomon Islands to Vanuatu, these data may prove useful in an improved understanding of mosquito biology that impacts the control of malaria.

## Additional files

Additional file 1: **Figure S1.** Twenty-four hour distribution of mosquito flight activity measured by infrared beam breaks of **a** *An. farauti* and **d** *An. hinesorum* females of the inseminated parous and inseminated nulliparous reproductive states under LD cycle conditions at minute resolution. A similar distribution shows **b** *An. farauti* and **e** *An. hinesorum* activity at hour resolution. Accumulation of mosquito activity over a 24 h time period of **c** *An. farauti* and **f** *An. hinesorum* females was calculated in a similar manner. Zeitgeber time (ZT) with ZT12 being the time of lights off, ZT10.5 the start of the 1.5 h dusk transition, and ZT0 occurring at the end of the 1.5 h dawn transition and 12 h after the onset of night. Day and night are indicated by horizontal white/black bars. (PDF 401 kb)

Additional file 2: **Figure S2.** Twenty-four hour distribution of mosquito flight activity measured by infrared beam breaks of *An. farauti* and *An. hinesorum* female regardless of reproductive state, so long as a reproductive state could be determined, under LD cycle conditions at **a** minute and **b** hour resolution. **c** Accumulation of mosquito activity over a 24 h time period of *An. farauti* and *An. hinesorum* females was calculated in a similar manner. Zeitgeber times as for Fig. 1 and Additional file 1: Figure S1 legends. Day and night are indicated by horizontal white/black bars. (PDF 245 kb)

Additional file 3: **Figure S3.** Analysis of the accumulation of flight activity over 24 h reveals differences between *An. farauti* complex mosquito species. Zeitgeber time at which each individual mosquito reached **a** 50% and **b** 75% accumulation of activity revealed a species-specific difference. Mean ± SEM (lines) and individual mosquito time points marking either 50 or 75% accumulation of individual daily activity (h) (*An. farauti*, blue circles; *An. hinesorum*, red squares). Mann-Whitney U-test was performed, *P < 0.05. (PDF 177 kb)

Additional file 4: **Figure S4.** Analysis of the timing of dusk/early night related onset of flight activity and peak of flight activity in *An. farauti* complex mosquitoes according to reproductive state and species. **a** Time of onset of activity, and **b** time of peak of activity. Median values (lines) and individual mosquitoes (*An. farauti*, circles; *An. hinesorum*, squares) in Zeitgeber time (ZT, h). Dusk transitions are indicated by horizontal white/black bars. (PDF 112 kb)

## Abbreviations

ZT: Zeitgeber time
LD: light:dark
DD: dark:dark
LAM: locomotion activity monitor
