# Supplementary figures and images for: Diel flight activity of wild-caught Anopheles farauti (s.s.) and An. hinesorum malaria mosquitoes from northern Queensland, Australia
Source: Parasit Vectors. 2019 Jan 22;12:48. doi: 10.1186/s13071-018-3271-0 (PMC6341630; doi:10.1186/s13071-018-3271-0)

Additional file 1: Figure S1

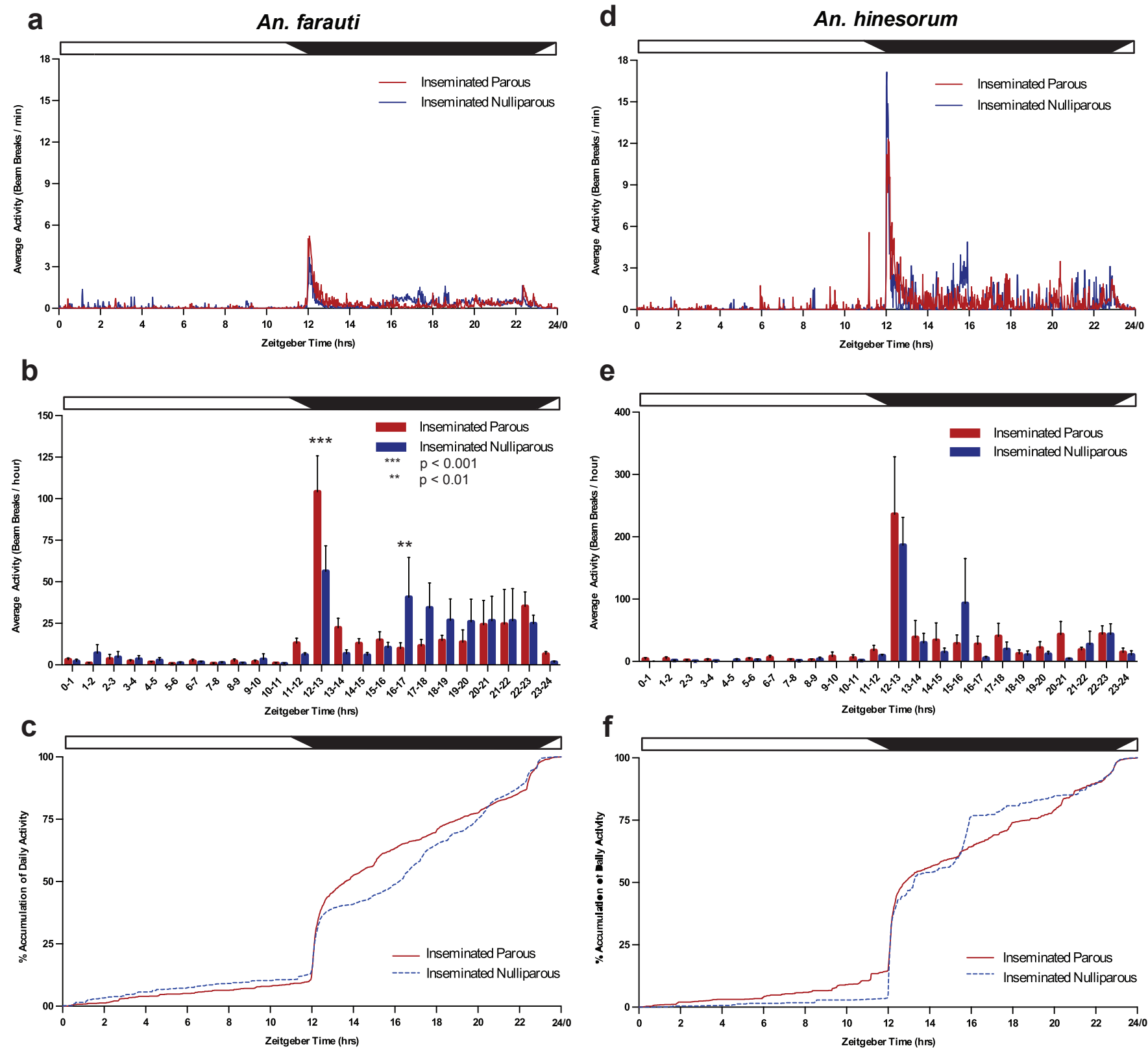

Supplement: Supplementary file 1 — Figure S1. Twenty-four hour distribution of mosquito flight activity measured by infrared beam breaks of a An. farauti and d An. hinesorum females of the inseminated parous and inseminated nulliparous reproductive states under LD cycle conditions at minute resolution. A similar distribution shows b An. farauti and e An. hinesorum activity at hour resolution. Accumulation of mosquito activity over a 24 h time period of c An. farauti and f An. hinesorum females was calculated in a similar manner. Zeitgeber time (ZT) with ZT12 being the time of lights off, ZT10.5 the start of the 1.5 h dusk transition, and ZT0 occurring at the end of the 1.5 h dawn transition and 12 h after the onset of night. Day and night are indicated by horizontal white/black bars. (PDF 401 kb) [file 13071_2018_3271_MOESM1_ESM.pdf]

## Additional file 2: Figure S2

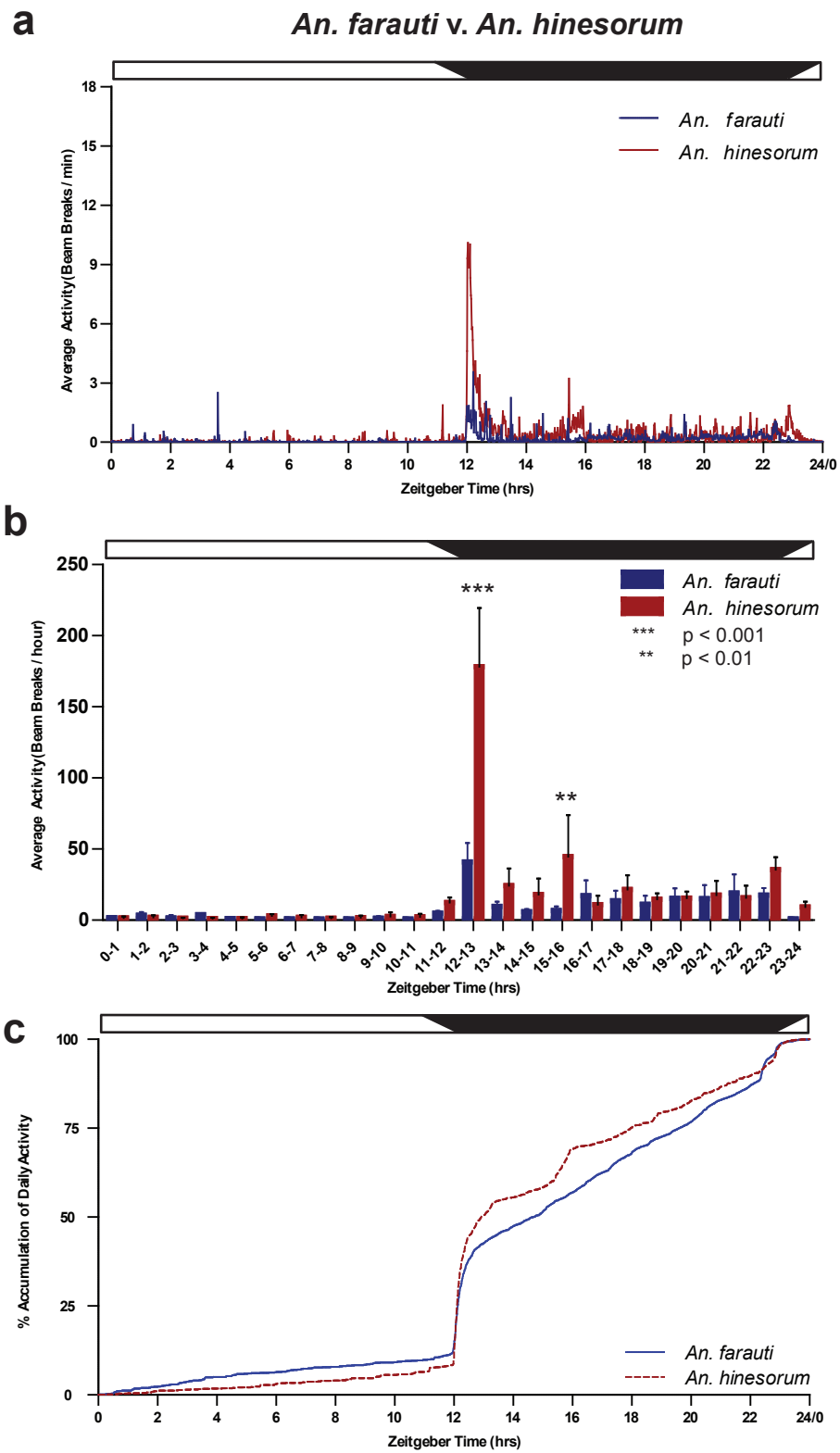

Supplement: Supplementary file 2 — Figure S2. Twenty-four hour distribution of mosquito flight activity measured by infrared beam breaks of An. farauti and An. hinesorum female regardless of reproductive state, so long as a reproductive state could be determined, under LD cycle conditions at a minute and b hour resolution. c Accumulation of mosquito activity over a 24 h time period of An. farauti and An. hinesorum females was calculated in a similar manner. Zeitgeber times as for Fig. 1 and Additional file 1: Figure S1 legends. Day and night are indicated by horizontal white/black bars. (PDF 245 kb) [file 13071_2018_3271_MOESM2_ESM.pdf]

Additional file 4: Figure S4

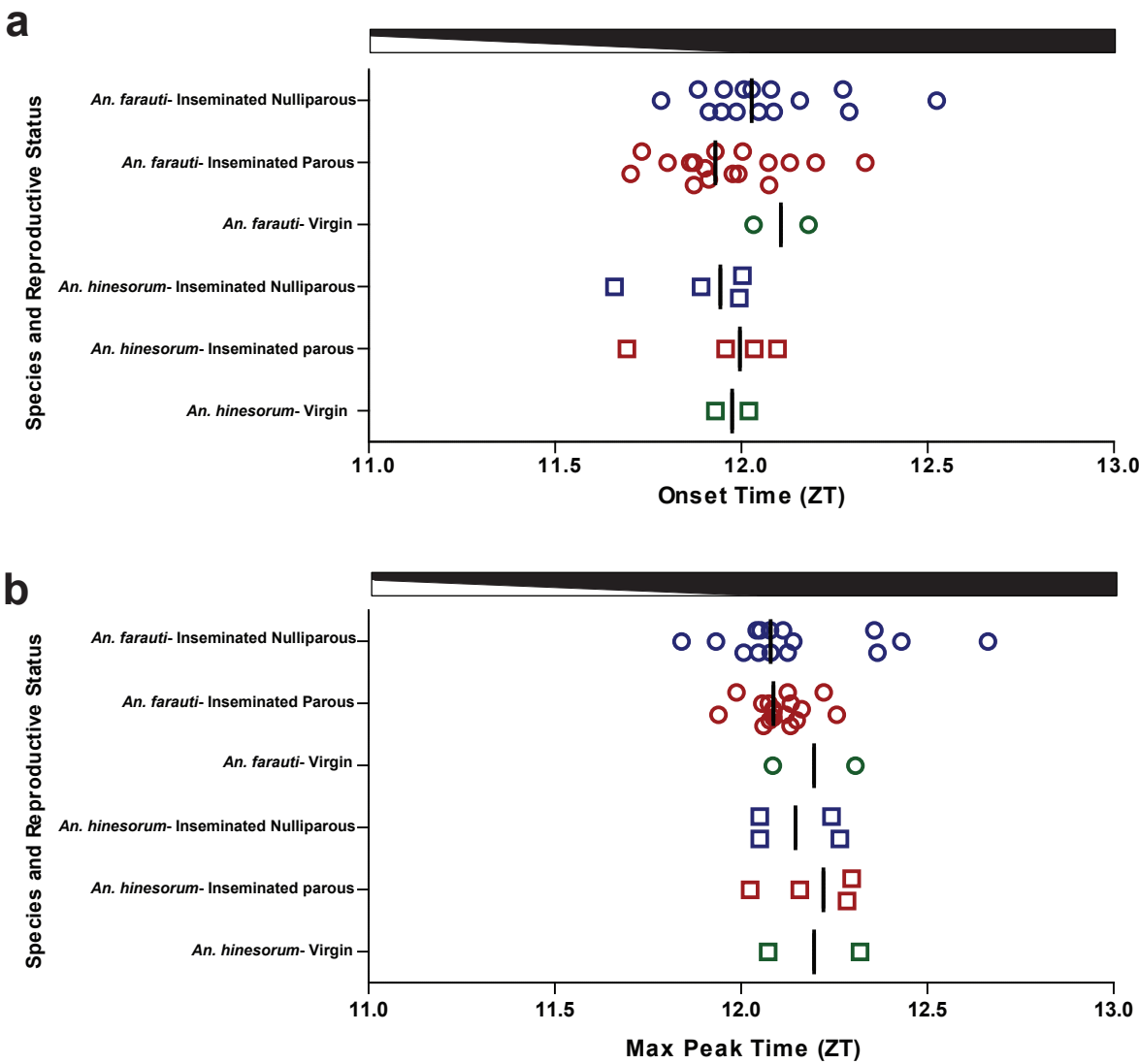

Supplement: Supplementary file 4 — Figure S4. Analysis of the timing of dusk/early night related onset of flight activity and peak of flight activity in An. farauti complex mosquitoes according to reproductive state and species. a Time of onset of activity, and b time of peak of activity. Median values (lines) and individual mosquitoes (An. farauti, circles; An. hinesorum, squares) in Zeitgeber time (ZT, h). Dusk transitions are indicated by horizontal white/black bars. (PDF 112 kb) [file 13071_2018_3271_MOESM4_ESM.pdf]
