# Supplementary material for: Diel flight activity of wild-caught Anopheles farauti (s.s.) and An. hinesorum malaria mosquitoes from northern Queensland, Australia
Source: Parasit Vectors. 2019 Jan 22;12:48. doi: 10.1186/s13071-018-3271-0 (PMC6341630; doi:10.1186/s13071-018-3271-0)

Additional file 3: Figure S3

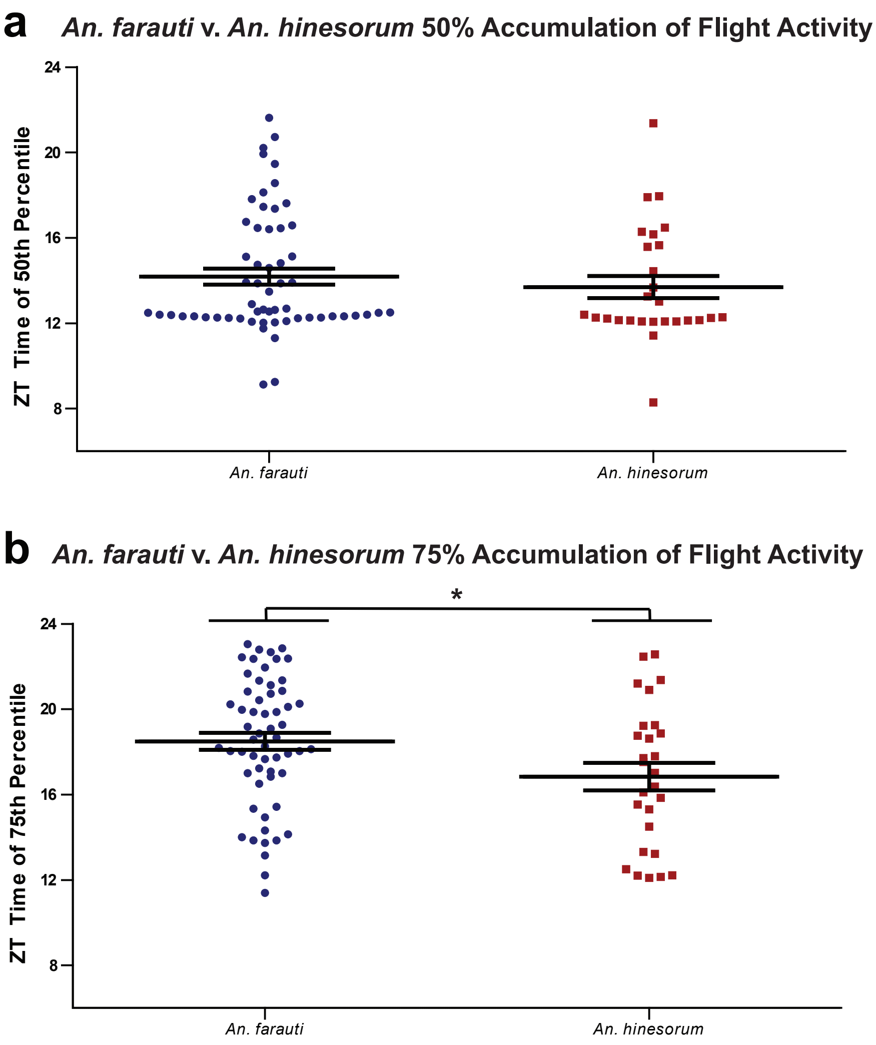

Supplement: Supplementary file 3 — Figure S3. Analysis of the accumulation of flight activity over 24 h reveals differences between An. farauti complex mosquito species. Zeitgeber time at which each individual mosquito reached a 50% and b 75% accumulation of activity revealed a species-specific difference. Mean ± SEM (lines) and individual mosquito time points marking either 50 or 75% accumulation of individual daily activity (h) (An. farauti, blue circles; An. hinesorum, red squares). Mann-Whitney U-test was performed, *P < 0.05. (PDF 177 kb) [file 13071_2018_3271_MOESM3_ESM.pdf]
